# Attrition and performance of community college transfers

**DOI:** 10.1371/journal.pone.0174683

**Published:** 2017-04-13

**Authors:** Lovenoor Aulck, Jevin West

**Affiliations:** Information School, University of Washington, Seattle, Washington, United States of America; University of California San Francisco, UNITED STATES

## Abstract

Community colleges are an important part of the US higher education landscape, yet the aptitude and preparedness of student transfers to baccalaureate institutions is often called into question. Examining transcript records and demographic information of nearly 70,000 students across over 15 years of registrar records at a public university, this study performed a descriptive analysis of the persistence, performance, and academic migration patterns of community college transfers, transfers from four-year institutions, and freshmen entrants. We found little difference between community college transfers and freshmen entrants in terms of post-transfer grades and persistence. Transfers from four-year institutions had higher grades but also had higher attrition rates than their peers. This study also found no strong evidence of transfer shock on students’ post-transfer grades. When examining the tendencies of students to shift fields of study during their educational pursuits, the academic migration patterns of transfer students were more concentrated than those of freshmen entrants.

## Introduction

The role of community colleges in the United States (US) higher education landscape has long been debated, particularly with respect to transferring students’ educational attainment. Part of this debate has centered on whether community colleges play a democratizing role by serving as low-cost university alternatives which increase access to post-secondary education (democratization) or have a negative effect on long-term student success and stunt educational pursuits (diversion). Arguing in favor of democratization, Rouse used instrumental variables with the High School and Beyond Longitudinal dataset (HSB) to find that a closer proximity to community colleges increases educational attainment without likely affecting the likelihood of receiving a bachelor’s degree [1]. Adding to this, Rouse then analyzed the relationship between the number of postsecondary institutions available to individuals and educational attainment to find that while going to a community college may slow the educational pursuits of some, these negative effects will be outweighed by the number of students who will benefit from attending community colleges [2]. Building upon this, Leigh and Gill argued that attending community colleges tends to increase educational attainment by between 0.4 and 1 years [3] while substantially expanding the educational aspirations of students [4].

Focusing on demographic differences, Gonzalez and Hilmer found that Hispanic community college attendees tend to complete nearly 2.6 years more college than similar non-attendees, with attending community college having no effect on Hispanic students’ attainment of a baccalaureate degree [5]. These views are countered by those advocating in favor of diversion, with both Clark [6] as well as Brint and Krabel [7] arguing that the education programs of community colleges inhibit students’ completion of four years of college. More recently, Alfonso used the National Education Longitudinal Study [8], Doyle used propensity score matching on the 1996 Beginning Postsecondary Students Dataset [9], Long and Kurlaender examined students in the State of Ohio’s public education system [10], and Lockwood Reynolds used the National Education Longitudinal Study of 1988 [11] to find that attending community college decreases educational attainment and the likelihood of completing a four-year degree.

In addition to the democratization versus diversion debate, the transfer function of community colleges has continually been called into question [12]. On one hand, it has been argued that the transfer function stands at the very foundation of what community colleges are intended to be. For example, Cohen and Brawer note that academic transfer preparation was among the most common curricular functions of community colleges noted by state legislatures [13]. That said, many have questioned the purpose of the transfer function and the need to have it, as noted by Doughtery [14]. Others still, such as Townsend [15], have advocated redefining how we view the transfer function altogether in light of changing community college transfer patterns, not the least of which is students switching between two- and four-year institutions (dubbed “swirling” [16]).

The perception of community colleges is further muddled by a lack of consensus regarding the post-transfer performance of students who transfer from community colleges to four-year institutions, both in terms of grades and graduation rates. Avakian et al. examined students at the University of Missouri-St. Louis [17] and Doucette and Teeter examined groups of students at 25 institutions in the state of Kansas (19 community colleges and 6 state universities) across 5 years [18], with both finding that community college transfers had higher attrition rates than freshmen entrants after transferring to a baccalaureate institution. Peng and Bailey also determined that transfer students had lower academic performance than freshmen entrants in their first years post-transfer after accounting for students’ backgrounds, individual characteristics, and financial aid status on the 1972 National Longitudinal Study [19]. Similarly, Porter later found that transfer students tended to have both lower graduation rates and lower grades compared to freshmen entrants at four-year schools when examining students from a single university across six years while controlling for a host of variables [20]. In contrast, Al-Sunbul found no difference in performance between the same student groups when examining 120 students (60 transfer and 60 freshmen) at the University of Nebraska-Lincoln [21]. Lee et al. developed logistic regression models using data on over 2,300 students in the HSB to determine that community college transfers and freshmen entrants did not differ in terms of graduation rates and entry to graduate school upon graduation [22]. It should also be noted that among Doucette and Teeter’s chief findings was no difference in grades between transfers and freshmen entrants, despite the difference in attrition rates [18].

The performance of community college transfers at baccalaureate institutions also brings the idea of “transfer shock” into focus. Hills first coined the term transfer shock to refer to a post-transfer drop in student performance during the first quarter/semester of enrollment, finding that transfer students suffer a drop in grades upon transfer and are less likely to graduate than freshmen entrants [23]. Around the same time, Knoell and Medsker also found evidence of transfer shock in a study of over 7,000 transfer students [24]. In opposition to this, Nickens argued that accounting for adequate academic variables during statistical analyses can explain transfer shock with respect to students’ grades, showing this with over 900 students at Florida State University [25] while Cejda later contended that the extent of transfer shock varies by discipline, even finding the performance of students in some disciplines improved after transfer (referred to as “transfer ecstasy”) [26]. In reviewing the available literature on transfer shock, Diaz found a lack of consensus on the matter, with 13 of 62 studies (about one-fifth) finding evidence of either transfer ecstasy or no transfer shock and 49 of 62 (about four-fifths) reporting evidence of transfer shock with varying degrees of recovery therefrom [27].

These divides in the perceived aptitude of the transfers from community colleges (and, in a broader sense, the value of the community college system as a whole) are particularly relevant in today’s educational and political climate with community colleges being viewed as a countermeasure to the enrollment caps and rising costs of tuition at four-year institutions. Additionally, an increased government focus on community colleges, including 2009’s American Graduation Initiative, which centered on an increase in the US’s investment in community colleges by billions of additional dollars (see http://bit.ly/18cFmVu), and America’s College Promise Act of 2015, which seeks to waive community college tuition for eligible students (see http://bit.ly/1CpCOSj and http://bit.ly/1NAQB73), has made it imperative that the risks, benefits, and costs for students attending two-year institutions are well understood. Furthermore, the uncertainty regarding the capabilities of community college transfers begs the question of whether these students are of lower aptitude than freshmen entrants or might be unfairly stereotyped as risky ventures [27].

In light of these issues, this study sought to examine differences in student performance among three groups of students at a large, publicly-funded, four-year state university in the United States: students who entered the university as freshmen, students who transferred to the university from community colleges, and students who transferred to the university from another four-year institution. We mined transcript and demographic data from university registrar databases to create a comprehensive student dataset of tens of thousands of students and compared the baccalaureate attainment and post-transfer performance of students across these three groups. From this, we present a descriptive analysis of the data that highlights the similarities and differences of these groups as they’ve progressed through a large, four-year state university. We do not draw any casual assertions about our findings but instead seek to present a descriptive look at students’ academic success at a large university using data that is routinely collected at institutions of higher education, thus highlighting how student transcript records can be leveraged for this type of institutional analysis. Our research aims are to examine the post-transfer success of college transfers in terms of persistence (as measured by graduation rates), performance (as measured by grades), and academic migration patterns (as measured by the movement of students from one field of study to another). The nearly 70,000 students observed in this study comprises one of the most expansive looks at transfer student performance (for both community college and baccalaureate transfers) and overall student persistence/attrition in the literature to date. We also believe this to be the among the first studies in which students’ paths through their baccalaureate education were traced at a large scale, giving a better sense of undergraduates’ academic migration patterns.

In so doing, we present a study in which we find that transfers from 2-year colleges and freshmen entrants have no meaningful difference in performance and attrition. Transfers from other baccalaureate institutions, meanwhile, have higher attrition rates but also perform better than their peers. No strong evidence for transfer shock was found when looking at the first few quarters of students’ enrollment at the university. Student migration patterns indicated that transfer students tend to have more concentrated/focused academic paths than their peers who entered the university as freshmen; we hope to build on this examination of migration patterns to better understand student attrition and university interconnectedness.

## Materials and methods

### Data collection

De-identified, pseudonymised student data was gathered from the University of Washington (UW) registrar’s databases in the summer of 2013 with the UW Administration’s consent. The data was gathered from the registrar databases, placed on a physical drive to be carried to our research lab, and then moved into an SQL database on a secure cloud server. The data was then curated and organized to allow for linking across tables and datasets. This process included clarifying coded variables with the data stewards, removing duplicated/missing values, and handling incorrectly entered data/typos. The process of requesting approval for the data, gathering the data, storing the data on a server, and curating the data took about two calendar years to complete.

The data included over 15 years of student records across all graduate, professional, and undergraduate degree programs at UW. This included, but was not limited to, students’ complete transcript records, students’ demographic information, and students’ degree records. It should also be noted that student financial aid data was not available for use from the university; accordingly, this analysis does not take into account students’ financial standing in any way, which is a large part of the student attrition puzzle. Transcript records consisted of course-level records for each course taken by each student and included time at which the course was taken, grade received, and declared majors when completing the course. Demographic information consisted of student-level information on gender, age, entrance exam scores, prior schooling, race/ethnicity, and resident status. For this study, data was restricted to all matriculated, degree-seeking undergraduate students who enrolled at the university between the years 1998 and 2006, inclusive, as either freshmen or transfers from US two- and four-year colleges (N = 69,118). Only undergraduate classes taken by students as matriculated students were included in the transcript data used in this study, which ultimately consisted of nearly 2,387,000 unique records of students taking classes.

**Fig 1:**
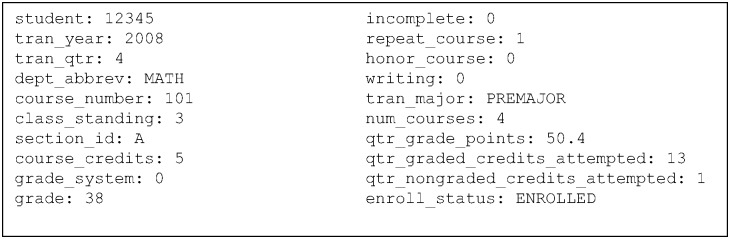
Sample transcript record. This provides a snapshot of the information available for each class taken by each student. Note that the enrollment status only indicates whether a student eventually withdrew from classes before the end of a single quarter—it was not used to determine graduation from the university.

A sample transcript record is provided in Fig 1 for reference. Aggregate measures (i.e. quarters enrolled, GPA, credits taken) were calculated using these individual records. Visiting and exchange students were excluded from the dataset as were students enrolled at the UW’s satellite campuses (the University of Washington Bothell and the the University of Washington Tacoma). In all, about 51.9% of the students in the dataset had first enrolled as freshmen (referred to as “freshmen entrants”), 25.6% were transfers from community colleges (referred to as “2-year transfers”), and 22.5% were transfers from colleges/universities that granted baccalaureate degrees (referred to as “4-year transfers”). About 96% of 2-year transfers were from public community colleges in Washington State while an additional 1% were from the State of California.

Students were also categorized in the registrar’s databases based on four demographic categories: self-described gender (male, female, or unidentified), self-described race (African American, American Indian, Asian, Caucasian, Hawaiian & Pacific Islander, or unidentified), self-described Hispanic ethnicity (Hispanic or not Hispanic/indicated), and resident status (resident or non-resident). Each student was categorized into only one of the above options for each demographic category and the categorization was mutually exclusive. Counts of students across student demographics are shown in Table 1.

**Table 1:** Demographic overview of students in dataset. Percentages indicate demographic composition across rows.

	All Groups	Freshmen Entrants	2-year Transfers	4-year Transfers
Total	69,118	35,855	17,680	15,583
Female	36,671 (53.1%)	19,074 (53.2%)	9,315 (52.7%)	8,282 (53.1%)
Male	32,401 (46.9%)	16,758 (46.7%)	8,360 (47.3%)	7,283 (46.7%)
Unidentified	46 (0.1%)	23 (0.1%)	5 (0.0%)	18 (0.1%)
African American	2,011 (2.9%)	1,017 (2.8%)	534 (3.0%)	460 (3.0%)
American Indian	938 (1.4%)	480 (1.3%)	245 (1.4%)	213 (1.4%)
Asian	16,037 (23.2%)	9,600 (26.8%)	3,067 (17.3%)	3,370 (21.6%)
Caucasian	39,442 (57.1%)	20,123 (56.1%)	10,292 (58.2%)	9,027 (57.9%)
Hawaiian/Pac. Islander	448 (0.6%)	265 (0.7%)	85 (0.5%)	98 (0.6%)
Unidentified	10,242 (14.8%)	4,370 (12.2%)	3,457 (19.6%)	2,415 (15.5%)
Hispanic	2,981 (4.3%)	1,485 (4.1%)	798 (4.5%)	698 (4.5%)
Not Hispanic/indicated	66,137 (95.7%)	34,370 (95.9%)	16,882 (95.5%)	14,885 (95.5%)
Residents	61,290 (88.7%)	30,510 (85.1%)	16,558 (93.7%)	14,222 (91.3%)
Non-Residents	7,828 (11.3%)	5,345 (14.9%)	1,122 (6.3%)	1,361 (8.7%)

### Defining non-completions

To determine attrition rates, a non-completion was defined as a student who did not successfully earn a baccalaureate degree from the university within a six-year period from when they first began coursework at the university (defined by when they first received a grade on their transcript). Successfully earning a baccalaureate degree was defined as the student being listed with a completed undergraduate degree in the registrar’s graduation records. The UW uses a quarter system of enrollment with 4 quarters (Autumn, Winter, Spring, and Summer) per year and students typically enroll for 3 quarters per year while not taking classes in Summer. Thus, a six-year time to completion is equivalent to 24 academic quarters from the student’s first quarter. All attrition rates are presented based on 6-year times to completion. Defining non-completions in this manner does not take into account the number of quarters a student was enrolled during the allotted period but rather the number of calendar years (measured in terms of academic quarters) since they first received a transcript grade. When presented annually, non-completions and graduates are grouped by the year in which they first started at the UW (i.e. by entering year cohort).

For transfer students, adjusted attrition rates were calculated by dividing the number of credits transferred to the university by the number of credits required for graduation with a baccalaureate degree (consisting of 180 credits) within 12 quarters of enrollment (corresponding to four years of full-time enrollment). This number of quarters completed outside the university was then subtracted from each student’s allotted time-to-degree to get adjusted times to completion. For example, a student transferring to the university with six quarters of credits completed at another institution will have 18 calendar quarters to graduate (not 18 quarters of enrollment to graduate) before being deemed a non-completion. It is understood that defining non-completions in this manner is very much institution-centric: it does not take into account student success before transferring to or after leaving the institution of interest but does take into account students who leave and return [28]. As such, this view of attrition does not account for students graduating from other institutions and should therefore be understood to be an *institution-centric attrition rate* and not a *student-centric graduation rate*.

### Analysis

Student grade point averages (GPAs; on a 4.0 scale) were calculated based on all undergraduate-level classes in which students received a numeric grade (early withdrawals, hardship withdrawals, and pass/fail classes were disregarded) as a matriculated student. The quarters in which students were enrolled were determined based on quarters in which students received at least one transcript grade (including non-numeric grades). Attrition rates were calculated by collating transcript data and using the above definition of non-completion in conjunction with the university’s database on degrees awarded. For students pursuing multiple undergraduate degrees, only the first degree awarded was taken into account when determining graduates and non-completions. For example, a freshmen entrant pursuing multiple degrees but only completing one in a 6-year period was deemed a graduate.

Transfer shock was examined by comparing students’ cumulative grades through each of the first three quarters of enrollment to their cumulative grades thereafter. Only students whose total credit count after each of these periods was equivalent to averaging at least full-time enrollment and had at least one subsequent quarter of full-time enrollment thereafter were included in this analysis. Previous studies on transfer shock examined the pre-transfer and post-transfer grades of students; however, due to institutional variability with respect to students’ previous schooling, this analysis focused only on student performance upon entry to the university.

In addition to looking at student performance, we believed that students’ patterns of movement and migration through their academics could provide additional insights into the attrition process, much like Chen examined with STEM fields [29]. As part of this work, we provide a preliminary look at the movement of students as they progress through their academics through the lens of field of study shifts—the migration of students from one academic field/disciple to another. The UW has three types of majors: open (those which can be declared freely by any undergraduate), minimum requirement (those that can be declared freely after certain course/grade requirements are met), and competitive (for which students must compete for a limited number of spots). Most entering students start with a pre-major designation until officially declaring a major typically in their sophomore or junior years of study. To examine academic migration patterns, all majors declared by graduating undergraduates during their academics at the university were grouped based on field of study. For example, the art, art history, and digital arts majors were all grouped into the “Art” field of study while all offerings from the university’s business school (e.g. accounting, marketing, and finance) were grouped into the “Business” field of study. Students’ transitions across fields of study were determined by using transcript histories to aggregate all majors declared for each student. After excluding all pre-major declarations, the count of students who shifted from each field of study to every other field of study was determined by chronologically examining each student’s transcript records and majors declared. For instances where a student had declared two or more concurrent majors (e.g. double majors), only the first major that was chronologically declared was used in the analysis. If that same student then dropped the earliest major designation, the second earliest of the declared majors would then also be included. For example, a student who declared a math major only to move into geography and then declared an economics major along with the geography major would only have the shift from math to geography included. If that same student was then to remove the geography declaration but keep the economics declaration, the shift from geography to economics would also be considered. It should also be noted that students’ majors were extracted from transcript records and, as such, were only reported on a per-quarter basis. Instances where students switched majors within the same field of study were disregarded. Majors that were not a natural fit with any other field of study grouping and had few instances of students shifting into/away from them were also disregarded, as were majors that were deemed too broad to be grouped into a specific field of study (i.e. the humanities and social science evening degree programs). A complete list of field of study groupings (including excluded majors and their shifts in/out) can be found in S3 Table in the supplementary material. Results from this analysis are presented as heatmaps showing the shifts in majors. Rates are also calculated to compare the in- and out-flow of students to and from different fields of study.

Tests on statistical significance were not reported as they would be superfluous with the samples sizes used in this study. Even minute differences between groups tend to be statistically significant when dealing with tens of thousands of observations. Instead, this study focused on the social and practical significance of the results from the data. As mentioned previously, the analysis presented is descriptive in nature—analytic models were not constructed and covariates were not adjusted for in the analysis. We understand this to be a departure from previous studies examining transfer students but we did not intend to draw causal inferences when our method of analysis (mining registrar database data) is limited in scope.

## Results

### Demographics

Table 2 shows differences between freshmen entrants, 2-year transfers, and 4-year transfers upon entry to the university. Age information was available for all students except 7 freshmen entrants. As expected, freshmen tended to enter the university at about 18 years old, which corresponds to the age at which US high school students typically finish their secondary education. Transfers from 2-year and 4-year institutions tended to be about 24 and 21 years old, respectively, when starting their academics at the university. SAT and ACT scores were available for 28,067 and 7,748 students, respectively (16,491 freshmen entrants, 3,127 2-year transfers, and 8,449 4-year transfers for SAT; 4,627 freshmen entrants, 815 2-year transfers, and 2,306 four-year transfers for ACT). Freshmen entrants and 4-year transfers had small differences in their entrance exam scores. 2-year transfers, meanwhile, had lower SAT and ACT scores than their peers. A demographic breakdown of ages at entry and entrance exam scores is provided in S1 and S2 Tables, respectively, in the supplementary material.

**Table 2:** Students at entry to university (Mean ± SD).

	All Groups	Freshmen Entrants	2-year Transfers	4-year Transfers
Age at Entry (yrs)	20.41 (± 4.44)	18.37 (± 0.70)	23.98 (± 5.75)	21.05 (± 4.93)
SAT Math Score	600.17 (± 88.11)	605.82 (± 85.35)	557.94 (± 89.29)	604.77 (± 88.78)
SAT Verbal Score	576.11 (± 94.01)	577.76 (± 91.74)	548.23 (± 99.35)	583.21 (± 94.54)
ACT Score	25.19 (± 4.21)	25.38 (± 4.15)	23.45 (± 4.31)	25.42 (± 4.15)

### Student attrition

Attrition rates are presented in Table 3. The adjusted 6-year attrition rates for 2-year transfers (23.5%) was slightly higher than freshmen entrants (20.9%) while that of 4-year transfers was more than 6 percentage points higher than both groups (Cohen’s D = 0.57 and 0.72 when comparing freshmen with 2-year transfers and 4-year transfers, respectively; calculated using a binary response variable). Additionally, for every demographic group, the attrition rates of 4-year transfers was higher than that of freshmen entrants and 2-year transfers, with the exception of non-residents, where the attrition rate of freshmen entrants was slightly higher than that of 4-year transfers. Interestingly, 4-year transfers had substantially higher attrition rates for males than females—while the differences in attrition rates between males and females was 1–2 percentage points for both freshmen entrants and 2-year transfers (with males having the higher rates), the difference in attrition rates between males and females for 4-year transfers was over 8 percentage points.

**Table 3:** Attrition rates by demographic. All rates are 6-year attrition rates adjusted for credits transferred. The Cohen’s D values for all freshmen vs all 2-year transfers and all freshmen vs all 4-year transfers were 0.57 and 0.72, respectively.

	All Groups	Freshmen Entrants	2-year Transfers	4-year Transfers
All	23.5%	20.9%	23.5%	29.6%
Female	22.2%	20.2%	23.1%	25.8%
Male	25.1%	21.7%	24.1%	34.0%
Unidentified	13.0%*	8.7%*	0.0%*	22.2%*
African American	31.9%	29.0%	29.4%	41.3%*
American Indian	34.3%	34.0%	29.0%	41.3%
Asian	22.3%	17.9%	25.6%	31.8%
Caucasian	23.2%	21.1%	23.4%	27.7%
Hawaiian/Pac. Islander	31.7%*	31.7%*	29.4%*	33.7%*
Unidentified	23.8%	22.7%	20.7%	30.3%
Hispanic	27.5%	26.3%	25.6%	32.5%
Not Hispanic/Indicated	23.4%	20.7%	23.4%	29.5%
Residents	23.0%	19.3%	24.0%	29.7%
Non-Residents	27.7%	29.9%	16.1%	28.5%

*—less than 500 students in demographic group

**Fig 2:**
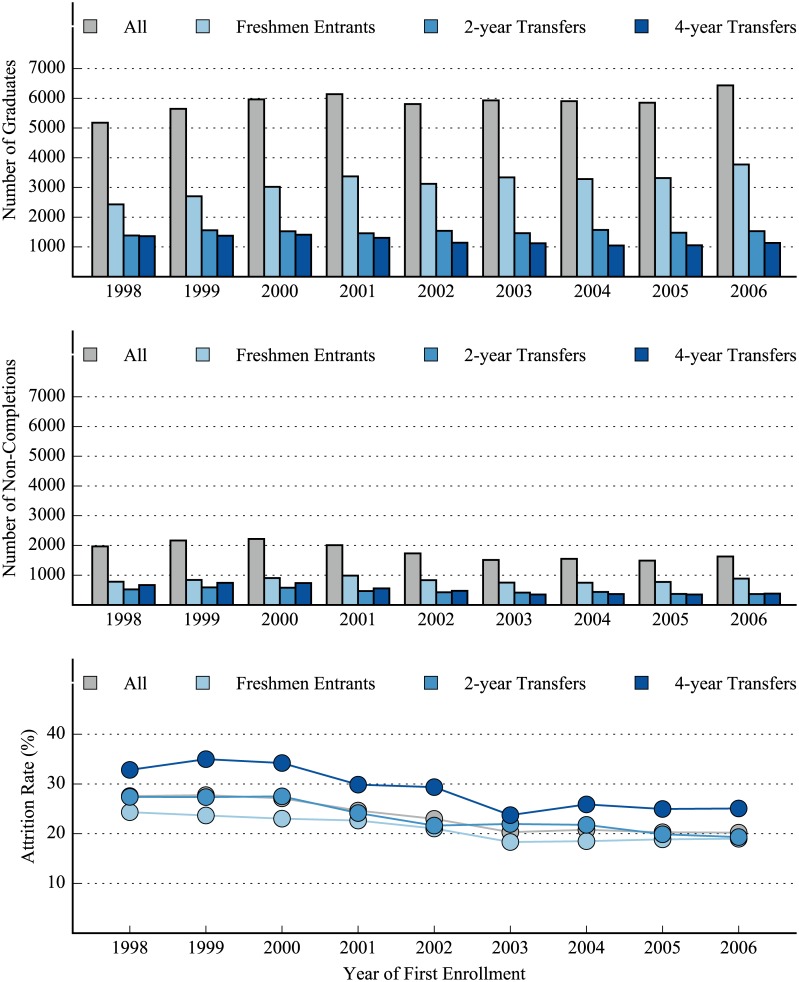
Graduate counts (top), non-completion counts (middle), and attrition rates (bottom) for all students, freshmen entrants, 2-year transfers, and 4-year transfers across time. An interactive visualization that allows for the data to be further segmented can be found at http://coursector.org/#/viz/attrition.

Attrition rates, counts of graduates, and counts of non-completions across time are shown in Fig 2. The years on the x-axis represent students’ first year at the university and counts/rates are calculated by entering year cohort. The attrition rates for all three groups followed similar trends, with all peaking between 1998–2000 and generally declining thereafter. The attrition rates for 4-year transfers remained higher than their peers for each year, with 32.82% of students in the 1998 entering class leaving the university before completing a baccalaureate degree and 25.07% of students in the 2006 entering class doing the same. The highest attrition rate seen for 4-year transfers was for the entering class of 1999 (34.98%) while the lowest was for the 2003 entering class (23.73%). Freshmen entrants, meanwhile, saw their highest attrition rate for the 1998 entering class (24.32%) and saw their lowest for the entering class of 2003 (18.32%) while attrition rates for 2-year transfers peaked for the 2000 entering class (27.51%) and saw a low for the 2006 entering class (19.31%). In fact, the average difference in attrition rates for freshmen entrants and 2-year transfers across the last three entering classes (2004–2006) was only 1.5 percentage points while that for the first three entering classes (1998–2000) was a more substantive 3.7 percentage points. Meanwhile, the attrition rates for 4-year transfers was an average of 6.6 percentage points higher than 2-year transfers for the first three entering classes and an average of 5.0 percentage points higher than 2-year transfers for the last three entering classes.

The overall attrition rate for the university trended downward for the period observed, declining from 27.56% for the entering class of 1998 to 20.20% for the entering class of 2006. Counts of total students (graduates + non-completions) for each of the three groups were also fairly stable over time. An interactive version of Fig 2 can be found at http://coursector.org/#/viz/attrition.

**Fig 3:**
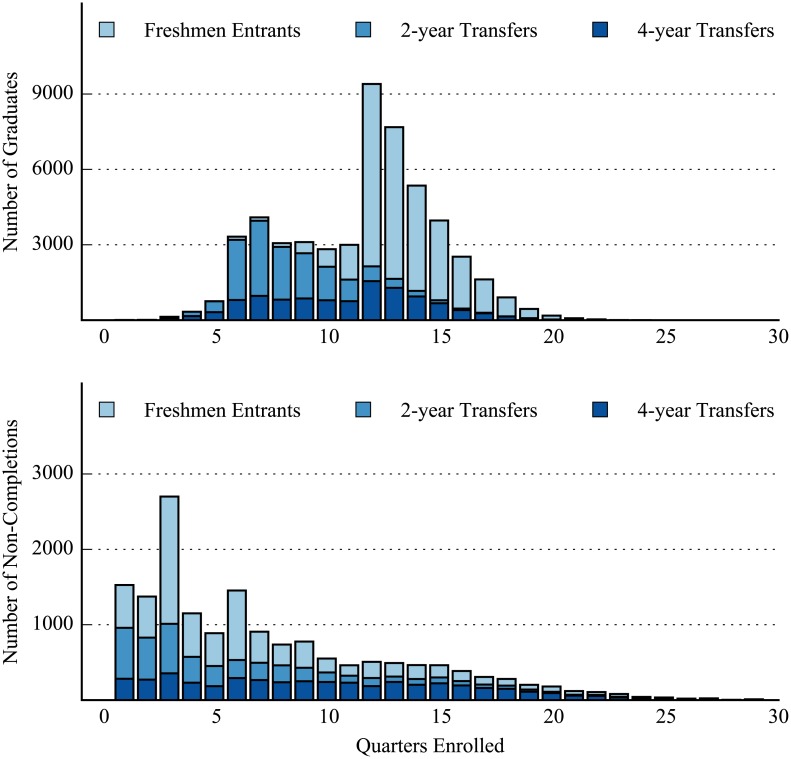
Number of quarters enrolled for graduates (top) and non-completions (bottom). Histograms look at all quarters student received some transcript grade. Mean, median, and standard deviations for graduates—Freshmen Entrants: 13.48, 13, 2.21; 2-year Transfers: 8.31, 8, 2.33; 4-year Transfers: 10.87, 11, 3.50. Mean, median, and standard deviations for non-completions—Freshmen Entrants: 6.80, 5, 5.33; 2-year Transfers: 5.79, 4, 4.87; 4-year Transfers: 9.84, 9, 6.10.

The distribution of the number of quarters enrolled by all graduates was distinctly bi-modal as shown in Fig 3 (note the trend of all graduates in the stacked bar plot and not the trend for any particular group). The first mode of the distribution peaked between 6–8 quarters of enrollment and is predominantly composed of 2-year transfers. It should be noted that 6 quarters corresponds to 2 years of enrollment while enrolling in 3 quarters per year (as would happen with a break in the summer) and 8 quarters of enrollment corresponds to 2 years of enrollment while taking 4 quarters per year. In all, 2-year transfers who graduated averaged 8.31 (SD: ± 2.33) quarters of enrollment before graduating. The second mode in the data occurs at 12–13 quarters of enrollment (corresponding to about 4 years of full-time coursework) and consists mostly of freshmen entrants, who enrolled for an average of 13.48 (SD: ± 2.21) quarters before graduating. 4-year transfers who graduated, meanwhile, showed more variability in the amount of time for which they enrolled before graduating, having typically enrolled for between 6 to 15 quarters and an average of 10.87 (SD: ± 3.50) quarters before earning their degree.

The distribution of the number of quarters enrolled by non-completions was right-skewed with a long right tail and a peak at 3 quarters of enrollment. A closer look at this peak revealed that 16.6% of non-completions stopped their coursework after exactly 3 quarters of enrollment while 34.4% of non-completions exited the university system after enrolling for less than 4 quarters (i.e. within their first academic year of enrollment). Of the three groups, 2-year transfers had the highest percentage of non-completions with less than 4 quarters of enrollment (45.4%), while freshmen entrants and transfers from 4-year institutions had rates that were lower (37.4% and 19.7%, respectively). A similar pattern persisted when looking at the percentages of non-completions who exited the university system within 1 calendar year of first enrollment (which were 31.9% of all non-completions), with transfers from 2-year institutions having the highest percentage (40.9%), followed by freshmen entrants (36.9%) and 4-year transfers (15.7%).

### Student performance

There were no meaningful differences in GPAs between freshmen entrants and 2-year transfers schools overall and when comparing graduates and non-completions of each group. Graduates and non-completions from the two groups had GPA differences of only a few hundredths of a grade point, as shown in Table 4. Interestingly, despite their higher attrition rates, 4-year transfers had GPAs higher than the other groups, with GPAs at least 0.1 grade points higher for graduates and at least 0.2 grade points higher for non-completions. As can be expected, non-completions had much lower GPAs than their graduating peers.

**Table 4:** Student cumulative grade point averages (max 4.0).

		All Groups	Freshmen Entrants	2-year Transfers	4-year Transfers
Mean ± SD	All	3.14 (± 0.56)	3.12 (± 0.56)	3.10 (± 0.58)	3.22 (± 0.55)
	Graduates	3.29 (± 0.38)	3.27 (± 0.37)	3.25 (± 0.39)	3.39 (± 0.36)
	Non-completions	2.64 (± 0.77)	2.57 (± 0.78)	2.59 (± 0.81)	2.81 (± 0.68)
Median	All	3.26	3.24	3.21	3.35
	Graduates	3.33	3.31	3.29	3.45
	Non-completions	2.74	2.65	2.70	2.89
25th %ile	All	2.89	2.88	2.84	2.97
	Graduates	3.05	3.04	3.00	3.18
	Non-completions	2.17	2.06	2.12	2.38
75th %ile	All	3.53	3.50	3.49	3.61
	Graduates	3.57	3.54	3.53	3.66
	Non-completions	3.22	3.17	3.20	3.31

When looking at students’ first year of enrollment at the university, no strong evidence of transfer shock could be seen. As shown in Table 5, students’ cumulative grades after their first single, first two, and first three quarters (excluding all students who did not average full-time enrollment across each period) were compared to their cumulative grades across all subsequent quarters (excluding students without at least one subsequent quarter of full-time enrollment). When comparing students’ first quarter of enrollment to subsequent quarters, students’ GPAs tended to decline on average after the first quarter, albeit slightly, thereby exhibiting evidence of transfer ecstasy. However, when looking at students’ performance after their first two quarters of enrollment, their grades tended to slightly improve thereafter, providing some evidence of transfer shock. This increase is even greater when comparing the first three quarters of enrollment to subsequent quarters. These trends were fairly consistent for each of the three groups. Transfers from 2-year colleges showed the greatest magnitude of transfer ecstasy and transfer shock. It should be noted, however, that the magnitude of these differences were only a few hundredths of a grade point at their highest.

**Table 5:** Student grade point averages as related to transfer shock (max 4.0; Mean ± SD). Note that sample sizes change across groups due to the selection criteria: only students whose total credit count after each of these periods was equivalent to averaging at least full-time enrollment and had at least one subsequent quarter of full-time enrollment thereafter were included in this analysis. Terms refer to terms at the university and not at students’ previous institutions.

	All Groups	Freshmen Entrants	2-year Transfers	4-year Transfers
Number of Students	47,748	26,300	10,752	10,696
GPA For Term 1	3.206 (± 0.56)	3.192 (± 0.56)	3.174 (± 0.56)	3.275 (± 0.57)
GPA After Term 1	3.194 (± 0.53)	3.178 (± 0.53)	3.157 (± 0.53)	3.269 (± 0.51)
Number of Students	54,400	31,530	11,060	11,810
GPA Through Term 2	3.186 (± 0.51)	3.169 (± 0.50)	3.160 (± 0.51)	3.254 (± 0.53)
GPA After Term 2	3.195 (± 0.53)	3.176 (± 0.53)	3.175 (± 0.52)	3.266 (± 0.52)
Number of Students	51,116	29,745	10,279	11,092
GPA Through Term 3	3.208 (± 0.47)	3.196 (± 0.45)	3.180 (± 0.46)	3.266 (± 0.49)
GPA After Term 3	3.223 (± 0.51)	3.207 (± 0.50)	3.206 (± 0.50)	3.284 (± 0.51)

### Student academic migration

**Fig 4:**
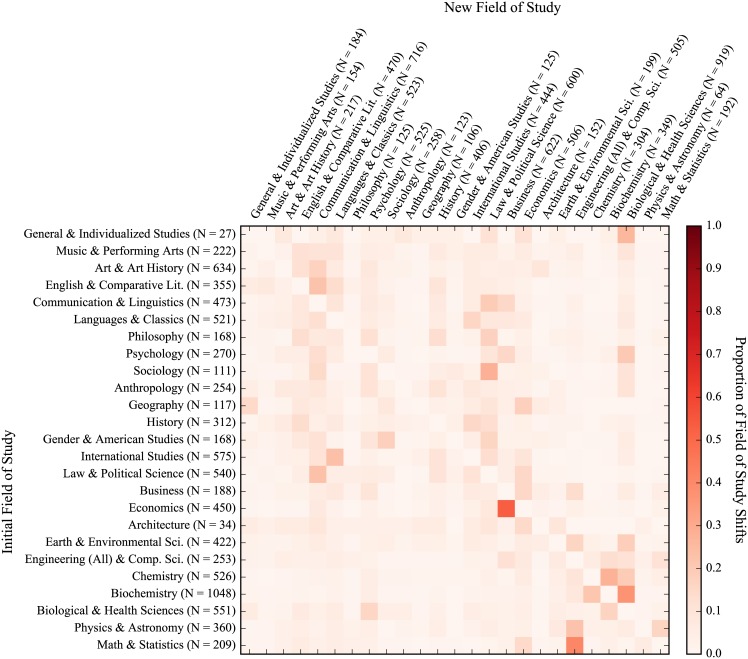
Field of study migration heatmap for all students. Values represent transfer of students from each row to each column. Proportions are normalized across rows. Counts for rows indicate outgoing students while counts for columns indicate incoming students. (NOTE: Figure rotated 90 degrees clockwise.)

**Fig 5:**
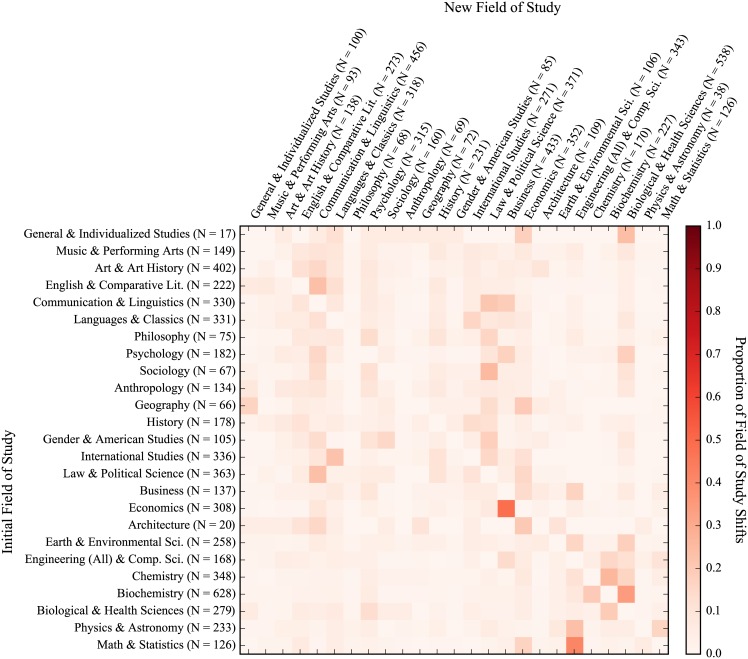
Field of study migration heatmap for freshmen entrants. Values represent transfer of students from each row to each column. Proportions are normalized across rows. Counts for rows indicate outgoing students while counts for columns indicate incoming students. (NOTE: Figure rotated 90 degrees clockwise.)

**Fig 6:**
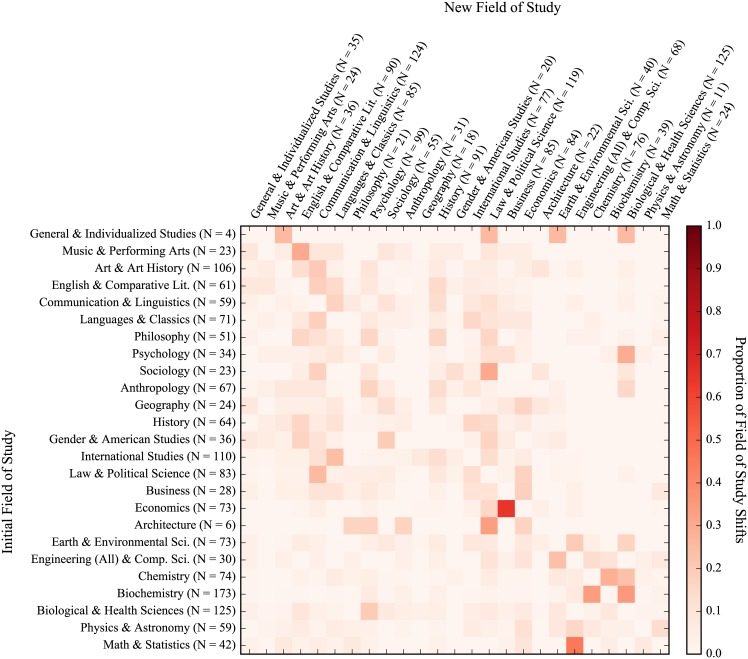
Field of study migration heatmap for 2-year transfers. Values represent transfer of students from each row to each column. Proportions are normalized across rows. Counts for rows indicate outgoing students while counts for columns indicate incoming students. (NOTE: Figure rotated 90 degrees clockwise.)

**Fig 7:**
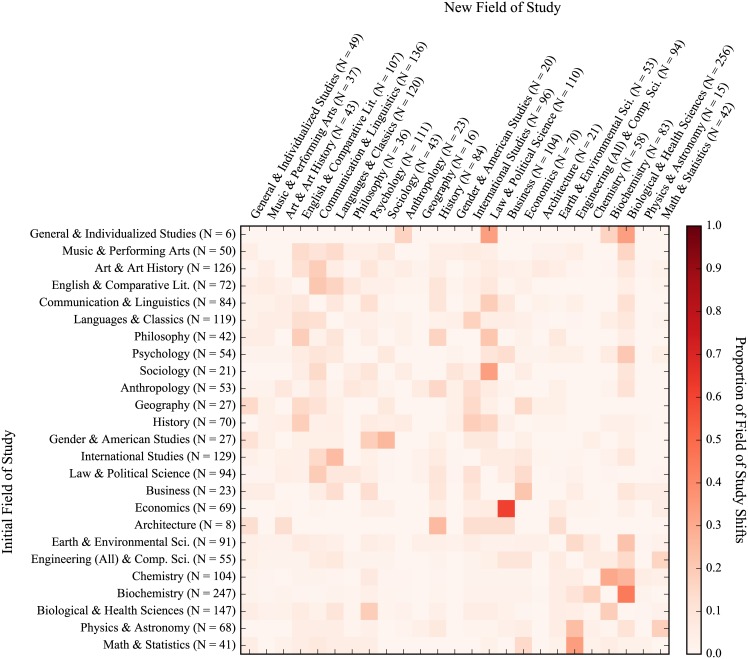
Field of study migration heatmap for 4-year transfers. Values represent transfer of students from each row to each column. Proportions are normalized across rows. Counts for rows indicate outgoing students while counts for columns indicate incoming students. (NOTE: Figure rotated 90 degrees clockwise.)

Heatmaps for the academic migration of all students, freshmen entrants, 2-year transfers, and 4-year transfers can be seen in Figs 4, 5, 6 and 7, respectively. The heatmaps are normalized across rows (i.e. across the source field of study of the shift) and reflect the tendency of graduating students to shift from one general field of study to another. Counts are provided across rows (outflux) and columns (influx). The fields are also subjectively arranged from less to more STEM-oriented fields along the rows and columns. As such, transitions near the diagonal represent less extreme shifts than those in the upper-right and lower-left corners. In all, 65,481 instances of students changing majors were seen with 8,788 unique shifts across fields of study (16.6 shifts per 100 students). As can be seen from visually inspecting the heatmaps of the migration patterns of the three groups, the movement of freshmen entrants was the most diffuse (i.e. had fewer areas of high migration traffic) while those of 2-year and 4-year transfers were similarly concentrated. Freshmen also had the highest frequency of shifts (19.3 shifts per 100 graduating students), with 4-year transfers and 2-year transfers having less shifts per graduating student (16.7 per 100 graduating students for 4-year transfers and 11.1 per 100 graduating students for 2-year transfers). This is very much reflective of the time taken by students from each group to graduate, as shown with Fig 3. In general (and as can be expected), students who have a longer time to graduate also tend to change their majors more often. However, when accounting for time, 4-year transfers had the highest rate of shifts per quarters enrolled (1.53 shifts per 100 quarters enrolled), with freshmen (1.43 shifts per 100 quarters enrolled) and 2-year transfers (1.33 shifts per 100 quarters enrolled) having slightly lower rates.

We also looked at the academic migration patterns of non-completions but found no striking patterns of shifts in students’ studies due to the sparsity with which non-completions shift. This is in part because pre-majors are excluded from this analysis and a large number of non-completions attrite as pre-majors. That said, as with rates of shifts over time for graduates, 4-year transfers did have a higher rate of shifts for non-completions than their peers (12.8 shifts per 100 students and 1.30 shifts per 100 quarters enrolled), which were much higher than than their peers who were freshmen (3.7 shifts per 100 students and 0.54 shifts per 100 quarters enrolled) and 2-year transfers (5.6 shifts per 100 students and 0.97 shifts per 100 quarters enrolled). Interestingly, these rates are lower than those for graduates on both a per student and per quarter basis, which could very well be reflective of the fact that most non-completions left the university rather early.

When looking at the migration patterns of graduates, 2-year transfers displayed several unique patterns with respect to the ratio of outflux to influx of students. Firstly, 2-year transfers had a larger ratio of outflux to influx for math & statistics with 1.75 graduating students leaving the field for every entering student, while freshmen entrants and 4-year transfers had approximately the same number of students exiting as entering (indicated by an outflux to influx ratio of approximately 1). The same was also true of philosophy, where 2-year transfers had a ratio of outflux to influx more than twice that of their peers. Conversely, 2-year transfers saw approximately the same number of students entering and exiting the fields of chemistry and the biological & health sciences while freshmen entrants and 4-year transfers had much higher rates of outflux for chemistry but much lower rates of outflux for the biological & health sciences.

Overall, physics & astronomy had the greatest ratio of outflux to influx across all three student groups with an average of 5.62 students leaving for every incoming student. Anthropology, art & art history, and biochemistry also had high outflux to influx ratios across the three groups of students, seeing averages of 2.07, 2.92, and 3.00 students leave for each entering student, respectively. General & individualized studies, meanwhile, had the lowest ratio of outflux to influx across all three groups with an average of 6.80 students entering the field for every one leaving. Psychology, engineering & computer science, sociology, and business also had consistently low outflux to influx ratios across the three student groups, with average outflux to influx ratios of 0.51, 0.50, 0.43, 0.30 (which represent 1.94, 2.00, 2.32, and 3.31 students entering the fields for every one leaving, respectively).

In general, three predominant migration trends held across all three student groups. First, students from economics had a strong tendency to shift into business when they move away from economics. A similar trend can be seen with students moving from math & statistics towards engineering & computer science. In the case of both students transitioning from economics to business and students going from math & statistics towards engineering & computer science, students were leaving a competitive major (one for which they had to apply for acceptance) for another competitive major. It could very well be the case that this was motivated by some financial incentive—both destination fields of study (business and engineering & computer science) can be argued to provide students with more employable opportunities upon graduation than their more theoretical counterparts (economics and math & statistics). Another predominant trend was a shift by students studying biochemistry into the biological & health sciences. This shift may be more reflective of the similarity between the fields than a tendency of students to shift the focus of their education entirely, particularly with respect to students intending to pursue a post-graduate professional degree in the health sciences (i.e. medicine, pharmacy, and dentistry).

Finally, we also looked at the percentage of each group that graduated within each field and their graduating GPAs as a possible explanation for why 4-year transfers have higher rates of attrition and GPAs than their peers. In particular, we were interested in seeing if this was related to 4-year transfers disproportionately going into fields with higher attrition rates and generally higher grades. We found that all groups tended to graduate from each field with about the same proportions (generally within 1–2% of each other), except in the biological and health sciences, which accounted for 12.6% of all 4-year transfer graduates compared to 9.2% and 8.7% of graduates for freshmen and 2-year transfers, respectively. When examining GPAs of graduates, grades for 4-year transfers from chemistry and the biological and health sciences were higher than their peers; GPAs of 4-year transfers who graduated were at least 0.23 and 0.15 grade points higher than other graduates from the same fields, respectively.

## Discussion

This study sought to compare freshmen entrants, 2-year transfers, and 4-year transfers at a large, public, state-funded university in the US in a descriptive manner across three metrics: performance, persistence, and academic migration. The nearly 70,000 students in the dataset are believed to comprise one of the largest looks at student attrition, transfer student performance, and student academic migration in the literature to date. This work does not draw strong causal associations due to limitations with the data and instead presents a descriptive view of the differences between the three groups. We believe this type of descriptive work can be useful for unearthing larger trends within the data (such as differences in attrition rates at scale) and highlighting areas for potential further investigation (such as inferring causality regarding students’ decisions to shift fields of study).

The attrition rates for the university as a whole were found to be lower than recent estimates of national averages (estimated to be about 40%) [30] [31]. In examining the performance of the three groups, little difference was found between freshmen entrants and two-year transfers, both in terms of attrition and grades. Despite transfers from 2-year institutions having substantially lower entrance exam scores, this group of students had attrition rates less than 3% greater than freshmen entrants and GPAs within two hundredths of a grade point of freshmen entrants. In one vein, this can be taken to mean that entrance exam scores give little indication about students’ future post-secondary academic performance, a point set forth by Hiss and Franks [32]; alternatively, an argument can also be made that these two-year transfers entered college with lower academic preparation as indicated by the lower entrance exam scores and were supported in their early post-secondary education by 2-year colleges to a degree that their performance varied little from more apt students. We believe the results of this work provide no compelling evidence for either case.

Across all majors, the 2.65 percentage point difference in attrition rate between freshmen entrants and 2-year transfers is at the upper bound of that reported by Porter [20] and contrast the findings of Weiss [33], Knoell and Medsker [24], and Avakian et al. [17]. The rather inconsequential differences in GPAs between the two groups is much smaller than differences previously reported by Porter [20] and echoes findings by Palmer [34], Doucette and Teeter [18], and Al-Sunbul [21]. Because majors can differ in their average GPAs, more work needs to be done in isolating those majors where differences exist between freshman and community college transfers.

Transfers from 4-year institutions were found to have substantially higher attrition rates than their peers while also having higher grades. These findings contrast the sentiment of many previous works, including Hills, who lists several studies that indicate 4-year transfers perform better than 2-year transfers [23], and Heiser and Abbed, who found 2-year and 4-year transfers to perform similarly [35]. Attrition rates were also substantially higher for transfers from 4-year institutions across nearly all demographic categories examined. The specifics of this could not be elucidated further from the data and a lack of literature on the matter leads us to believe that understanding the performance of transfers from 4-year institutions warrants further investigation.

With regards to transfer shock, students’ early grades were compared to their subsequent grades at the same institution. This is very different than the approach of most previous studies examining transfer shock, which compared students’ pre-transfer GPA at their previous institution with their grades immediately after transfer [23] [27]. Comparing pre- and post-transfer GPAs is beneficial for understanding how student grades differ upon transfer and allowing institutions to adequately prepare students for potential declines in grades, thereby minimizing any discouragement on the part of the student. However, we believe that such an analysis should take into account institutional variability, especially when looking at students entering a large, publicly-funded university like the UW. In this work, we compared students’ early grade records with subsequent records at the same university. Though this approach gives no indication of differences between students’ pre-transfer and post-transfer performance, it does give an indication of any adjustment time required by students when entering a new institution, be it as freshmen or transfer students.

The data examined showed no strong evidence of transfer shock. Differences between students’ early grades at the university and later grades were only as small as a few hundredths of grade points at most, similar to many previous studies examining pre-transfer and post-transfer GPAs [27]. Though transfers from 2-year institutions did in fact suffer decreases in grades during the first two and first three quarters of enrollment, these dips in performance were found to be similar to those seen in the performance of freshmen entrants and transfers from 4-year institutions during their respective starts at the university. Furthermore, if only the first quarter is examined, the data shows evidence of transfer ecstasy and not transfer shock for all three groups.

In addition to examining student performance, we also believed that student migration patterns could provide additional insight into the attrition process. This study provides a preliminary look at both student migration patterns as well as the related interconnectedness of majors/departments within an academic institutions, which we hope to build off in our future work. When looking at student academic migration patterns, 2-year transfers had the fewest number of shifts across fields of study per student, while transfer students in general had more concentrated migration patterns than freshmen entrants. This could be a reflection of freshmen entrants’ uncertainty with regards to their educational pursuits while transfer students, especially those transferring more credits into the university, have more defined educational trajectories post-transfer. An example of this can be seen when looking at the number of fields of study that shift into the biological sciences for 2-year transfers (relatively very few) compared to same for the two other groups. 2-year transfers also showed a greater propensity to shift away from math & statistics and philosophy than their peers but no strong trends were evident when looking at the influx/outflux of students across similar fields of study (e.g. a greater propensity to leave the social sciences for STEM fields or vice-versa), regardless of their previous schooling.

A few strong trends persisted for all three groups, namely students shifting from economics into business, from math & statistics into engineering & computer science, and from biochemistry into the biological & health sciences. For the first two, these transitions indicate a shift of academic focus that may be motivated by the employability of the major as students are transitioning from more theoretical fields (i.e. economics and math & statistics) to those that teach more technical/practical skills (business and engineering & computer science). We also understand that some of these shifts may also be related to institutional requirements when students pursue a particular field of study including GPA requirements, applications, and departmental admissions policies. As such, a shift in major may not necessarily be reflective of students’ interest in other fields—it merely formalizes the pursuit of an alternate but similar major. As an example, a student may be interested in a particular major for which an application is required and decides to formally declare a major in another field of study as a placeholder while completing prerequisites. If this student is then rejected from their first-choice major and continues to pursue a degree in the major that had been initially declared as a placeholder, the intended pursuit of the major from which they were rejected cannot be tracked. We understand this to be a limitation with respect to using transcript data in this manner, as only formally declared majors are tracked.

The analysis techniques used in this study are mostly descriptive and should be easily replicable by those with access to similar data in university registrar databases. That, however, brings two related limitations of this study into focus: the fact that the study focused on descriptive results and that it centers on a view of attrition and performance that is from the perspective of information in university databases alone. Due to solely using transcript information in a single university, causal underpinnings of the performance of transfer students were not examined. We understand this to be a departure from previous studies looking at transfer students, which focus more heavily on econometric analyses and causal assertions; in part, we intended for this work to serve as a case study for how transcript-level data routinely collected can be used for institutional analysis at scale. Additionally, without longitudinal data across institutions or additional controls for student characteristics, it’s difficult to further explore the differences between the groups examined. As such, this study focused strictly on the post-transfer performance of transfer students. Because students were not tracked prior to their transfer to the university or after leaving the university, their pre-transfer and post-university performance is unknown. We understand this limits the degree to which a comprehensive view of student performance can be drawn. We also understand that it is very often the case that only the more capable and connected students transfer from a community college to a 4-year institution; as such, these post-transfer comparisons are not reflective of community college students more broadly. However, this has little effect from the perspective of the university and we believe that viewing our analysis from an *institution-centric perspective* rather than a *student-centric perspective* with respect to attrition and performance mitigates some of these concerns.

Additionally, we used transcript data alone to perform this analysis—no students were interviewed or surveyed due to the pseudonymised nature of the data and the sheer volume of students in the study. Sociological and individual factors related to attrition and dropout were not investigated. This study’s analysis did not seek to develop a new model related to student attrition but sought to further the commentary and discussion on the post-transfer success of students; as such, we focused on descriptive results as mined from a large, public university. Regardless of a lack of focus on causality and the limitations with using a data-mining-centric approach, we believe the provided results help further the commentary on transfer students in the American education system. We also believe we provide a unique view of the university academic system from the perspective of student migration across fields of study.

## Future work

As a next step in analysis, we would like to extend the descriptive analysis we present here and examine the differences between groups more rigorously. This includes unearthing causal interpretations while adjusting for covariates in the analysis across groups. Another potential avenue for furthering this work is tracing students from community college data through their time at baccalaureate institutions. Additionally, efforts are also under way to include data from additional universities to better understand student performance post-transfer while teasing out institution-specific effects. We are also developing models for predicting students who attrite from the university early and are using feature-engineering-based machine learning approaches to predict such students early in their academic careers [36]. We also intend to use the data we have compiled to examine the performance and persistence of students based on gender, ethnicity, and enrollment in first-year programs, among other attributes.

The work with academic migration patterns has laid the foundation for additional exploration of the interconnectedness of university systems and departments/fields of study. Stepping away from the siloed view of departments and majors, we believe examining the interconnectedness of fields by alternate means (e.g. using non-departmental classes taken by students from a particular department to link departments) can provide unique views of the university system not possible without large-scale, course-level data. Finally, we would like to use our future research in the educational space to demonstrate the effectiveness and power of mining data in registrar databases—data that is widely collected, curated, and stored across the world—to better understand processes underlying academic institutions and education as a whole. For information on additional projects, please visit www.coursector.org. Code for this project is available at https://github.com/lovenoor/plos_one_student_transfers.

## Conclusion

This study presents a view of transfer student performance from community colleges and 4-yr institutions. The results contribute to the debate surrounding the role of community colleges. We find that students transferring from 2-year colleges perform as well as native students post-transfer across a dataset spanning over 15 years of transcript records (1998–2013). Additionally, this study also sheds light on the issue of the performance of lateral transfers at baccalaureate institutions and questions as to why they may have stronger grades but also higher attrition rates than their peers. Lastly, this study presents information on student migration patterns, finding that freshmen tend to shift between majors at a greater rate than transfer students while also exhibiting more diffuse migration patterns.

## Supporting information

S1 Table Ages at Entry by Demographic (Mean ± SD). (TEX)

S2 Table Total SAT Score (Math + Verbal) by Demographic (Mean ± SD). (TEX)

S3 Table Groupings of Majors. Counts are given for majors excluded in analysis.
(TEX)
